# Correction of Severe Knees Valgus Deformities in a Patient With Renal Osteodystrophy

**DOI:** 10.5435/JAAOSGlobal-D-22-00113

**Published:** 2022-09-15

**Authors:** Cen Bytyqi, Dafina Bytyqi, Bujar Shabani, Venera Bytyqi, Nderim Salihaj

**Affiliations:** From the Department of Orthopaedics (Dr. C. Bytyqi, Dr. D. Bytyqi, Dr. Shabani, Dr. Salihaj), University of Prishtina “Hasan Prishtina,” Prishtina, Kosovo, and Medizinische Klinik 2 (Dr. V. Bytyqi), Friedrich-Alexander University of Erlangen-Nürnberg (FAU), Erlangen, Germany.

## Abstract

Renal osteodystrophy (ROD) is a complex and rare entity that refers to a large spectrum of abnormalities of skeletal homeostasis in patients suffering from chronic kidney disease. The goal of this study was to present the outcome of the very rare case of an adult with severe deformity of painful bilateral valgus knees due to ROD, requiring multilevel osteotomies above and below the knee. A 42-year-old male patient was admitted to our department with painful severe bilateral valgus knees deformity due to ROD. The patient underwent bilateral lateral opening-wedge osteotomy of distal femur and medial tibial closing-wedge osteotomy. The osteotomies site healed in 8 weeks without complications. The surgical treatment of lower limb valgus knee deformities secondary to ROD is a challenging and demanding procedure. In our patient, the femoral opening-wedge osteotomy with blade-plate fixation, and tibial closing-wedge osteotomy with plate fixation, restored almost normal knee congruency to prevent lateral unicompartmental degenerative deterioration of the knee.

The kidney has a notable role in bone metabolism, maintaining equilibrium of calcium and phosphate in blood and activation of vitamin D3. Renal osteodystrophy (ROD) is generally considered as a consequence of hyperparathyroidism, which occurs because of hyperphosphatemia and hypocalcaemia, due to impaired phosphate renal elimination.^1,2^ ROD is a complex entity that refers to a large spectrum of abnormalities of skeletal homeostasis in patients suffering from chronic kidney disease or end-stage renal disease.^3,4^ The treatment of valgus malalignment of the knees in patients with ROD remains a challenge, especially in adult nontreated patients. The goal of this study was to present the outcome of a rare case of an adult with severe and painful valgus knee deformity due to ROD, treated with multilevel osteotomies above and below the knee.

The ethical committee approved the study, and the patient signed an informed consent.

## Case Presentation

A 42-year-old male patient presented to our clinic with pain and progressive deformity in bilateral knees. The patient has had a medical history of end-stage chronic renal disease, and at the age of 24 years, he received a living-donor renal transplantation from his mother. Standard knee x-rays showed a severe valgus deformity of 28° on the right side and 30° on the left side and level II osteoarthritis on the lateral compartment of both knees (Figure 1). To manage the pain and the deformity, we proposed varus osteotomies in both legs.

**Figure 1:**
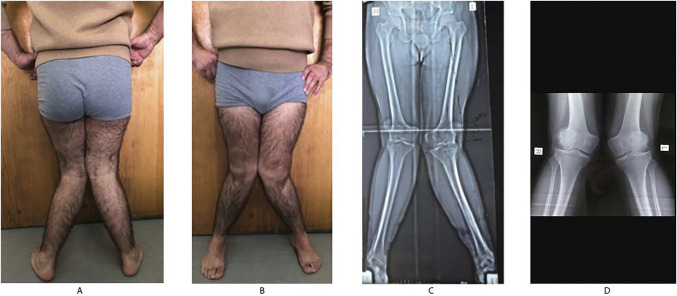
**A** and **B**, Clinical images showing the anterior and posterior pre-reconstruction. **C**, The preoperative standardized long leg standing panoramic radiograph of the lower limb was done for frontal plane analysis. The hip-knee-ankle angle on the right side was 28° and 30° on the left side. **D**, Short radiographs show the level II osteoarthritis on the lateral compartment of the knee.

### Preoperative Plan

The preoperative planning was based on a panoramic AP radiograph of the lower limb, with loading. The mechanical lateral distal femoral angle was 63° on the left side and 65° on the right side; the mechanical medial proximal tibial angle in our patient was 93° on the left side and 92° on the right side. The valgus deformity on the distal femur was 24° on the left side and 22° on the right side. Valgus deformity in the proximal tibia in our patient was 6° on both sides.

Based on these measurements and considering the magnitude of deformity, a femoral supracondylar opening-wedge osteotomy and tibial closing-wedge osteotomy were planned for both legs. The goal was to bring the hip‐knee‐ankle angle around 2° to 3°, the mechanical lateral distal femoral angle 86°, and the mechanical medial proximal tibial angle 86°.

Blade plate fixation of supracondylar femoral osteotomy with bone grafting taken from medial tibial wedge closing corrective osteotomy was planned. For fixation of the medial closing-wedge osteotomy of the tibia, a medial plate was planned.

The surgeries were programmed to be done separately on each leg at the 8-month interval. Because the deformity was more severe on the left side, the first surgery was done on the left leg, followed by the right 8 months later. It was aimed that this solution would provide adequate long-term stability and correct alignment.

### Surgical Technique

The patient was placed on the supine position on the radiolucent surgical table with an image intensifier under the table.

In the femur, the lateral approach was used. The iliotibial band was incised along the line of skin incision. Vastus lateralis was retracted anteriorly to expose the distal femur. To avoid fractures, the entry point of the blade chisel was prepared with an orthopaedic cannulated drill bit of 3.5 mm. This chisel advanced under image intensifier guidance on the frontal and sagittal plans in the supracondylar region. After the implant was inserted with the blade parallel to the joint line lateral, supracondylar femoral osteotomy was done. On the medial aspect of the proximal tibia, a direct longitudinal incision was made. Although the knee was fully extended, under C-arm visualization, the medial closing-wedge osteotomy was done and fixed with a medial tibial plate. The bone taken from proximal medial tibial osteotomy was used as a graft for femoral osteotomy. Partial weight-bearing using crutches was started 5 days postoperatively. The osteotomies site healed in 8 weeks without complications, and full weight-bearing was permitted (Figure 2).

**Figure 2:**
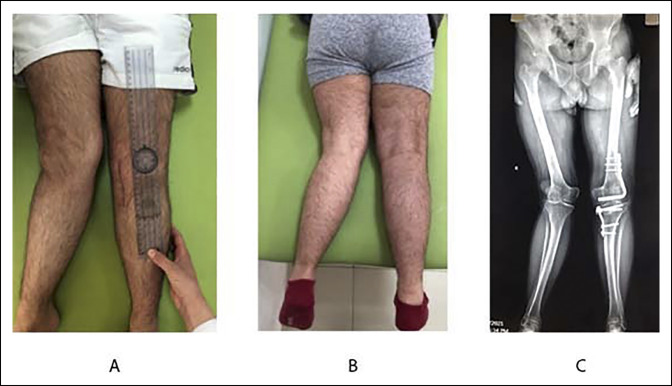
**A** and **B**, Photographs showing the clinical difference between the corrected left side and the untreated right side. **C**, The 7-month postoperative anterior-posterior radiograph showing knee deformity correction and complete healing of osteotomized bones.

Eight months later, the same operation with the same technique was done on the right side. One year after the operation, the patient had no functional problems in the activities of daily living and sports. The range of motion was 120/0/0° on the right knee and 125/0/0 on the left one. A whole‐leg standing radiograph showed normal limb alignment with a hip‐knee‐ankle angle of 3° (Figure 3).

**Figure 3:**
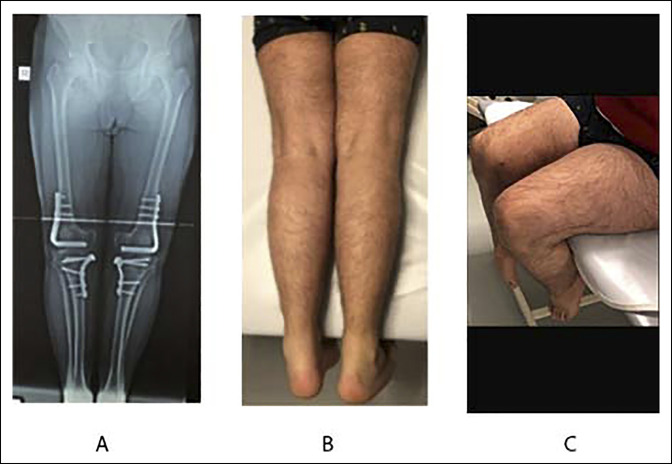
**A**, The anteroposterior radiograph showing the final standing long leg and (**B**–**D**) clinical and functional postoperative evaluation. The range of motion on the left knee was 0/0/125 and the range of motion on the right knee 0/0/120.

## Discussion

ROD due to secondary hyperparathyroidism is associated with numerous osteoarticular complications. The surgical treatment of the skeletal deformities due to ROD remains still a challenge. Previous studies have shown different approaches regarding the treatment of valgus knee deformities caused by ROD.^7^ Most of these studies show the correction of the deformities during childhood, usually with guided growth techniques. In the current report, we show a unique case of surgical treatment of a severe valgus knee deformity due to ROD in an adult patient. With valgus deformities at the knee, the joint line often slopes from superolaterally to inferomedially in the coronal plane, and the resultant deformity resides largely on the femoral side. Because of this, proximal tibial osteotomy creates an oblique joint line and is less successful in the management of valgus knees. On the other hand, varus-producing distal femoral osteotomies (DFOs) are a better option to treat lateral compartment osteoarthritis, especially if the deformity is greater than 12° of valgus.

We did most of the correction in the femur and used the blade plate because it proved to show adequate long-term stability in open supracondylar osteotomy especially in combination with bone grafting taken from proximal medial tibial osteotomy. Because of the severity of deformity, we had to add the tibial closing-wedge osteotomy to correct it.^8-10^ In recent years, the authors have increasingly suggested the use of varus-producing DFOs in young patients with isolated lateral compartment knee osteoarthritis. Ekeland et al^11^ evaluated 24 opening-wedge DFOs and concluded with 74% of survival rate at 10 years of follow-up. Forkel et al^12^ described 23 DFOs and reported a notable increase in all subitems of KOOS (Knee Injury and Osteoarthritis Outcome Score) with a closing-wedge technique.

## Conclusions

With careful preoperative planning and a meticulous surgical technique, distal femoral and proximal tibial varus osteotomy is a valuable option for the treatment of patients with severe symptomatic and knees valgus deformities in the setting of ROH.
